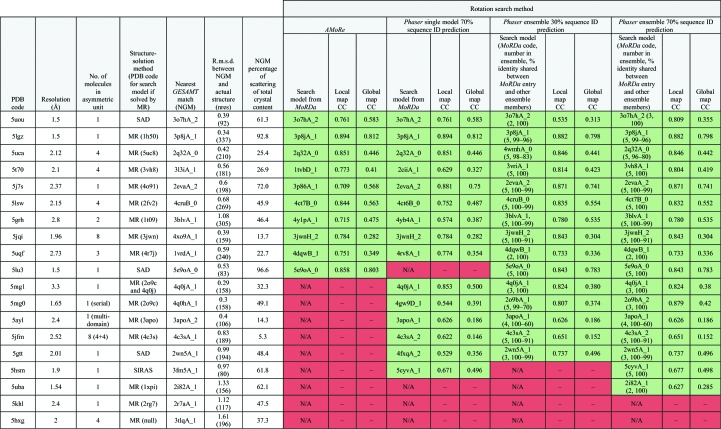# Using *Phaser* and ensembles to improve the performance of *SIMBAD*

**DOI:** 10.1107/S2059798319015031

**Published:** 2020-01-01

**Authors:** Adam J. Simpkin, Felix Simkovic, Jens M. H. Thomas, Martin Savko, Andrey Lebedev, Ville Uski, Charles C. Ballard, Marcin Wojdyr, William Shepard, Daniel J. Rigden, Ronan M. Keegan

**Affiliations:** a Institute of Integrative Biology, University of Liverpool, Liverpool L69 7ZB, England; b Synchrotron SOLEIL, L’Orme des Merisiers, BP 48, 91192 Saint Aubin, Gif-sur-Yvette, France; c STFC, Rutherford Appleton Laboratory, Harwell Oxford, Didcot OX11 0FA, England; d CCP4, Research Complex at Harwell, Rutherford Appleton Laboratory, Harwell Oxford, Didcot OX11 0FA, England; e Global Phasing Ltd, Cambridge CB3 0AX, England

**Keywords:** molecular-replacement pipeline, contaminants, structure solution, *SIMBAD*, ensembles, sequence independent

## Abstract

The conventional approach to search-model identification in molecular replacement (MR) is to screen a database of known structures using the target sequence. However, this strategy is not always effective, for example when the relationship between sequence and structural similarity fails or when the crystal contents are not those expected. An alternative approach is to identify suitable search models directly from the experimental data. *SIMBAD* is a sequence-independent MR pipeline that uses either a crystal lattice search or MR functions to directly locate suitable search models from databases. The previous version of *SIMBAD* used the fast *AMoRe* rotation-function search. Here, a new version of *SIMBAD* which makes use of *Phaser* and its likelihood scoring to improve the sensitivity of the pipeline is presented. It is shown that the additional compute time potentially required by the more sophisticated scoring is counterbalanced by the greater sensitivity, allowing more cases to trigger early-termination criteria, rather than running to completion. Using *Phaser* solved 17 out of 25 test cases in comparison to the ten solved with *AMoRe*, and it is shown that use of ensemble search models produces additional performance benefits.

## Abstract 2

Improvements to the sensitivity of the search for suitable molecular-replacement search models in *SIMBAD* through the use of *Phaser* and an ensemble-based model database are reported.

## 1. Introduction   

Molecular replacement (MR) remains the most popular method to solve the phase problem in macromolecular crystallography since it is quick, inexpensive and often highly automated (Evans & McCoy, 2008 ▸; Long *et al.*, 2008 ▸; Scapin, 2013 ▸). Conventional MR exploits the fact that evolutionarily related macromolecules tend to be structurally similar. Therefore, when correctly placed within the asymmetric unit of a target structure, a homologous protein can provide a sufficiently accurate approximation of the phases to solve the unknown structure (Rossmann, 1972 ▸, 1990 ▸). As evolutionarily related molecules are likely to have similar protein sequences, sequence similarity often provides a quick and easy route to identify suitable homologues for MR. However, search models selected by sequence similarity can give poor results for a number of reasons. These include cases where only distant, low-sequence-identity homologues can be identified, which can often be too structurally divergent from the target. Even where high-sequence-identity homologues are available, they may have crystallized in different conformational states and hence prove too structurally distinct to succeed. Another possibility is that a contaminant protein has crystallized in place of the target protein.

An alternative approach adopted by some developments is to perform a brute-force search of the entire PDB (Stokes-Rees & Sliz, 2010 ▸; Hatti *et al.*, 2016 ▸). *SIMBAD* (Simpkin *et al.*, 2018 ▸) provides a novel, sequence-independent method to identify search models for MR by performing a rotation-function search on every structure in the *MoRDa* database (Vagin & Lebedev, 2015 ▸) against the experimental diffraction data. As the scores from the rotation function for a suitable search model tend to be distinctly better than the scores from a poor search model, this provides an alternative route through which to identify suitable search models.

Here, we explore whether use of the maximum-likelihood fast rotation function implemented in *Phaser* (v.2.8.2; Read, 2001 ▸; Storoni *et al.*, 2004 ▸), instead of the Patterson-based fast rotation function in *AMoRe* (version adapted for use in *CCP*4; Navaza, 1987 ▸, 1993 ▸), can improve the success rate of *SIMBAD*. In the maximum-likelihood rotation function, a search model is sampled in rotational space and the orientation that predicts the data obtained with the highest probability is selected (Evans & McCoy, 2008 ▸). A key advantage of using a maximum-likelihood approach is that both experimental and search-model (coordinate) errors are explicitly modelled in the probability calculations, whereas Patterson methods assume that there are no errors (Evans & McCoy, 2008 ▸). By modelling these errors, likelihood methods tend to give clearer solutions in difficult cases (Read, 2001 ▸).

In *Phaser*, an initial root-mean-square deviation (r.m.s.d.) between the search model and target structure is typically estimated from the shared sequence identity between the two. *Phaser* will adjust this initial estimate using the variance r.m.s. (VRMS) parameter to help optimize its log-likelihood gain (LLG) calculation and thereby increase the chance of identifying a correct solution (Oeffner *et al.*, 2013 ▸). As the sequence of the target structure is unknown in *SIMBAD*, this study samples a low value (30%) and a high value (70%) of the sequence identity, which are converted into estimates of target versus search model structural error in a size-dependent fashion by *Phaser*.

In addition to using the *Phaser* rotation search, we have also explored the use of ensemble search models in *SIMBAD*. It has been shown that using search models containing multiple structures which have been clustered and aligned into ensembles can be more effective than using the individual structures (Leahy *et al.*, 1992 ▸; Pieper *et al.*, 1998 ▸; Chen *et al.*, 2000 ▸; Rigden *et al.*, 2002 ▸; Bibby *et al.*, 2012 ▸; Keegan *et al.*, 2015 ▸, 2018 ▸). In MR, the coordinates from a search model are converted into a set of calculated structure factors for comparison with the experimental data. In *Phaser*, ensembles allow the generation of a statistically weighted set of structure factors based on the variation in the ensemble (Read, 2001 ▸). This improves the signal-to-noise ratio in the likelihood function (McCoy *et al.*, 2007 ▸) and therefore also increases the chance of finding a correct solution. Here, the ensembles are derived using the alignment-truncation procedure implemented in *MrBUMP* (Keegan *et al.*, 2018 ▸).

We observe that the use of *Phaser* with an error estimate calculated from an assumed search-model sequence identity of 70% with the target sequence and ensemble search models significantly improves the ability of *SIMBAD* to identify a good search model in a set of 25 test cases that contained a wide range of resolution limits, numbers of copies in the asymmetric unit, space groups, monomer sizes and secondary-structure types (Simpkin *et al.*, 2018 ▸). Using the *AMoRe* method ten out of 25 cases were solved, while using the *Phaser* method 17 out of 25 cases were solved. Note that here ‘solved’ refers to the correct placement of a suitable search model but does not necessarily indicate that the MR solution could be used for successful model completion through automated model building.

## 2. Methodology   

The methodology for *SIMBAD* has been described in detail previously (Simpkin *et al.*, 2018 ▸). In outline, a lattice-parameter search is followed by the screening of a small database of common contaminants and then the *MoRDa* database using the *AMoRe* rotation function. These three elements can be run singly or sequentially.

### 2.1. Phaser   

**Figure 1:**
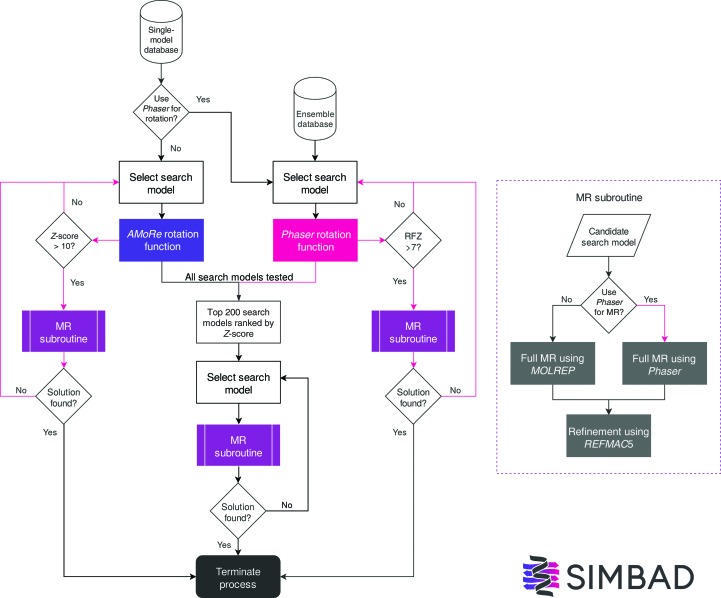
Flowchart detailing the new *Phaser* pathways (shown with pink arrows) that have been implemented in *SIMBAD*. Criteria for a solution are *R* values of <0.45 from *REFMAC*5 and/or an LLG of >60 and TFZ of >8 if MR is performed using *Phaser*. *SIMBAD* will use the MR subroutine to test the top 200 solutions ranked by *AMoRe* *Z*-score or *Phaser* RFZ score unless a solution has been identified by the early-termination procedure. The early-termination procedure is triggered when a search model achieves a *Z*-score of >10 in an *AMoRe* search or an RFZ of >7 in a *Phaser* search. This search model is tested using the MR subroutine and if the placed model meets the criteria for a solution then the process will terminate early.

*SIMBAD* was modified to run the *Phaser* likelihood-enhanced fast rotation function (MR_ROT mode) in the screening step of the pipeline (Fig. 1 ▸). The rotation likelihood function uses a Rice distribution, in which the effect of the estimated model error is accounted for by the σ_A_ term in the intensity-based LLG function (Read & McCoy, 2016 ▸). This method is most effective when the data are provided as intensities. Where amplitudes are provided, assumptions need to be made about how the intensities have been converted to amplitudes. When a sequence is known, *Phaser* can use this to estimate the error in the model (Oeffner *et al.*, 2013 ▸). As the true sequence identity is unknown in *SIMBAD*, the rotation search was tested using fixed values of 30% and 70%. The rotation-function *Z*-score (RFZ) produced by *Phaser* was used to rank the results, with the top 200 ranking search models carried forward to the full MR stage of the pipeline.

The MR subroutine in *SIMBAD* was modified so that it could be run with *Phaser* in addition to *MOLREP* (Fig. 1 ▸). Previously, it was run only through *MOLREP* (Vagin & Teplyakov, 2010 ▸). We reasoned that any advantage conveyed by the *Phaser* rotation function might require the running of *Phaser* in the full MR step to successfully identify a solution.

There is a trade-off to be made between the sensitivity of the search and the time it takes to process hundreds of search models in MR. To reduce the computational time, a 30 min time limit was imposed on each *Phaser* job and it was instructed to search for only a single copy of the search model. This strategy was based on the suggestions of Stokes-Rees & Sliz (2010 ▸) and Hatti *et al.* (2016 ▸), who observed that MR calculations on a single chain that take longer than 30 min rarely lead to correct solutions. Other than setting the run time and the sequence identities for the search model, *Phaser* was run using its default options for all of the test data sets. This included allowing *Phaser* to vary the resolution limit for the data used in the search.

In order to reduce the computational expense of the *Phaser* search, an early-termination function was implemented to test whether search models that had particularly high RFZ values might result in a solution in full MR and refinement, where the criteria used for a solution are (i) *R* values below 0.45 and/or (ii) an LLG and TFZ of over 60 and 8, respectively. The early-termination function is only triggered if the RFZ value in the rotation search exceeds a certain threshold. In our testing a conservative RFZ value of 10 was used as this threshold. However, we observed that an RFZ value of over 7 was typically indicative of the correct orientation of a good search model. Therefore, in the distributed version of *SIMBAD* a default threshold of 7 will be used.

Additionally, the improved Matthews coefficient (Kantardjieff & Rupp, 2003 ▸) implemented in the cell-content analysis module of *Phaser* was used to predict the molecular weight (MW) of the target structure prior to the rotation search. We reasoned that search models close to this predicted value would be more likely to give a solution. Therefore, search models were placed in ascending order using the equation
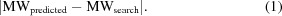



This increased the likelihood that a suitable search model would be tested early in the search and therefore results in earlier termination. However, at this time this method is only suited to crystal structures containing one molecule in the asymmetric unit. This is discussed further in Section 4.2.

### 2.2. Ensemble generation   

Ensembles were generated for each entry in the *MoRDa* database using the ensembling procedure described by Keegan *et al.* (2018 ▸). Here, the sequence from each *MoRDa* domain was used to identify suitable homologues from the PDB using *phmmer* (Eddy, 2011 ▸). *MrBUMP* includes a set of redundancy-reduced derivatives of the PDB sequence database for use in the *phmmer* search. For *SIMBAD*, ensembles are generated from the 100% database (*i.e.* no models are removed based on sequence redundancy).

*Phmmer* returns a list of matches with scores based on sequence alignment to indicate how similar they are to the target sequence. *MrBUMP* uses a *phmmer* score threshold of 20 to eliminate unrelated proteins and homologues that are likely to be too dissimilar to be used as suitable search models in MR. This score is constructed by inferring residue probabilities from a standard 20 × 20 substitution score matrix, plus two additional parameters for position-independent gap-open and gap-extend probabilities (for further details, see the *HMMER* user manual; http://eddylab.org/software/hmmer/Userguide.pdf). To construct each ensemble, we took a maximum of five structures matching our *MoRDa* entry. If no suitable homologues could be found, the original single model was used instead. This was the case for 2024 out of 81 716 entries in the *MoRDA* database. The database we created therefore contains a mixture of single structures and ensembles, and it will henceforth be referred to as the ‘ensemble database’ to distinguish it from the database containing only single structures.

Once a set of suitable homologues from the PDB has been selected, *MrBUMP* performs homologue modification to try and improve the chance of successful MR. This is performed by comparing the information provided by the alignment of the target sequence with the matching sequences found by *phmmer*. In this case the target sequence is that of the *MoRDa* domain. Modifications include the truncation of side chains and the trimming away of loops. This is performed using the *Sculptor* application (Bunkóczi & Read, 2011 ▸), which modifies the homologues based on the provided alignment.

The final step is to align the edited structures into an ensemble. This alignment is performed using *GESAMT* (Krissinel, 2012 ▸; Krissinel & Uski, 2017 ▸). Once aligned, *MrBUMP* puts the resulting ensembles through a truncation procedure to remove the more variable regions and thereby identify a structurally conserved common core.

For testing, an ensemble database was generated from the version of the *MoRDa* database released on 12 March 2016. This was the same version as used in the original testing. To ensure that the released structures relating to our test cases were not included in the ensembles, the sequence databases included with *MrBUMP* were modified to only include PDB entries released prior to February 2017.

## 3. Results   

### 3.1. Testing   

In previous work describing *SIMBAD* (Simpkin *et al.*, 2018 ▸), a test set of 25 structures that had recently been deposited was compiled to assess the ability of *SIMBAD* to solve novel structures. This test set contained a wide range of resolutions, copies in the asymmetric unit, space groups, monomer sizes and secondary-structure types.

Given that the true structures were known for our test cases, *GESAMT* (Krissinel, 2012 ▸; Krissinel & Uski, 2017 ▸) was used to identify the most structurally similar entries in the *MoRDa* database. Default options for *GESAMT* were used, including a requirement that the alignment of the target to the model covered at least 70% of both the target and the model. MR was performed using these structures to identify the maximum number of solutions possible. In 19 out of the 25 cases (76%) the *MoRDa* database contained sufficiently similar homologues to solve the target. Putative solutions identified by either an LLG of > 60 and a TFZ of >8 and/or *R* factors of <0.45 were verified using a map correlation coefficient (map CC) between the *F*_calc_ and φ_calc_ from the potential MR solution and the *F*_obs_ and φ_calc_ from the deposited model using *phenix.get_cc_mtz_pdb* (Adams *et al.*, 2010 ▸). The global map CC values ranged from 0.146 to 0.812, with an average of 0.46, and the local map CC values in the region of the search model ranged from 0.529 to 0.894, with an average of 0.762. A global map CC of ≥0.2 or a local map CC of ≥0.5 was considered to be indicative of success, with additional verification carried out through manual inspection.

#### 3.1.1. *AMoRe* using single search models   

In order to test whether *Phaser* would improve the performance of the screening step of *SIMBAD*, the performance of *SIMBAD* using *AMoRe* was tested first. Both the *AMoRe* and *Phaser* screening steps were paired with *Phaser* and *REFMAC*5 (Murshudov *et al.*, 2011 ▸) for full MR and refinement so that the rotation functions used in the screening step could be directly compared. Using *AmoRe*, ten out of the 25 test cases (40%) could be solved.

#### 3.1.2. *Phaser* using single search models   

One of the goals of this testing was to explore whether the likelihood-enhanced rotation search implemented in *Phaser* (McCoy, 2004 ▸) would improve the performance of *SIMBAD*. Preliminary tests using a fixed r.m.s.d. estimate of 0.5 Å showed that the error estimate supplied to *Phaser* was important to maximize the rotation score for a good search model. Oeffner *et al.* (2013 ▸) introduced a function to estimate an initial r.m.s.d. value from the percentage sequence identity and the size of the search model. This work showed that it was beneficial to increase the r.m.s.d. estimate with the size of the search model. We therefore employed a fixed sequence identity in place of a fixed r.m.s.d. to benefit from this function. A predicted sequence identity of 70% was employed to screen the single models in the *MoRDa* database. This choice led to the solution of 15 of the 25 test cases (60%), a significant improvement on the ten cases solved using the *AMoRe*-based method. Unexpectedly, a suitable search model could not be found for PDB entry 5lu3, a case that was solved in the *AMoRe* search.

#### 3.1.3. *Phaser* using ensembles   

A database of ensembles was generated, each derived from an entry in the *MoRDa* database. Given that *Phaser* generates a set of weighted structure factors based on the variation in an ensemble, we reasoned that ensembles might compensate for a poor initial estimate of r.m.s.d. by downweighting the more variable parts in our search models (Keegan *et al.*, 2018 ▸).

**Figure 2:**
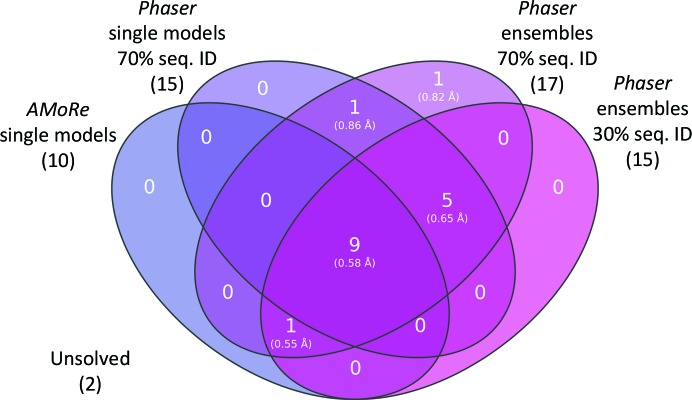
Venn diagram comparing the number of solutions and the average r.m.s.d. of successful search models obtained from the 19 test cases (known to be solvable) using the default *AMoRe* rotation search and the *Phaser* rotation search in several differing configurations. As mentioned in the text, two of the 19 cases were not solved in any run even when using the best available search model. Not shown here are the six cases that we were unable to solve using the closest available search model.

A high (70%) and a low (30%) predicted sequence identity were selected in order to sample alternative initial r.m.s.d. values. Using ensembles with the higher estimate yielded an additional two solutions; 17 of the 25 test cases (68%) were solved. The lower estimate was only able to match the single-model performance; 15 of the 25 test cases (60%) were solved. A comparison of these results is shown in Fig. 2 ▸, with additional details in Table 1 ▸.

Only two cases (PDB entries 5hxg and 5khl) remained that were known to be solvable using the best available homologues in the *MoRDa* database, but which were not solved by *SIMBAD* in any run. Given the importance of the error estimate in the likelihood-enhanced rotation search, the experiment was repeated using the true r.m.s.d. value between the targets and the best available search models (1.61 Å for PDB entry 5hxg and 1.12 Å for PDB entry 5khl). This gave maximum RFZ values of 4.52 and 4.29, respectively. These values fell below the noise level observed in a typical *SIMBAD* run and therefore it was unlikely that these search models would have been carried through into the full MR step. We further explored this by observing the rotation scores using sequence-identity values ranging from 10% to 100% in increments of 10%. However, this failed to improve upon the scores given above.

We attribute the poor rotation scores to the fact that the best available search models have r.m.s.d.s of 1.61 and 1.12 Å from the true structure and they constitute 37% (one domain from a two-domain dimer) and 47% (one of two domains) of the total scattering content of the target crystals. Both are also in high symmetry space groups, *P*3 (PDB entry 5hxg) and *P*4_3_22 (PDB entry 5khl), making the signal from the single domains in the rotation search relatively weak. Strategies for modifying *SIMBAD* to solve such cases are discussed later.

The average run times were measured for the *AMoRe* method and the *Phaser* method using 70% sequence identity and ensembles. Using a 100-core cluster (2.6 GHz, Intel Xeon E5-2640), the *AMoRe* method took an average of 10.2 h, whereas the *Phaser* method took an average of 27.8 h, although this would improve to an estimated 18.2 h using the updated early-termination function.

### 3.2. Comparative rankings   

**Figure 3:**
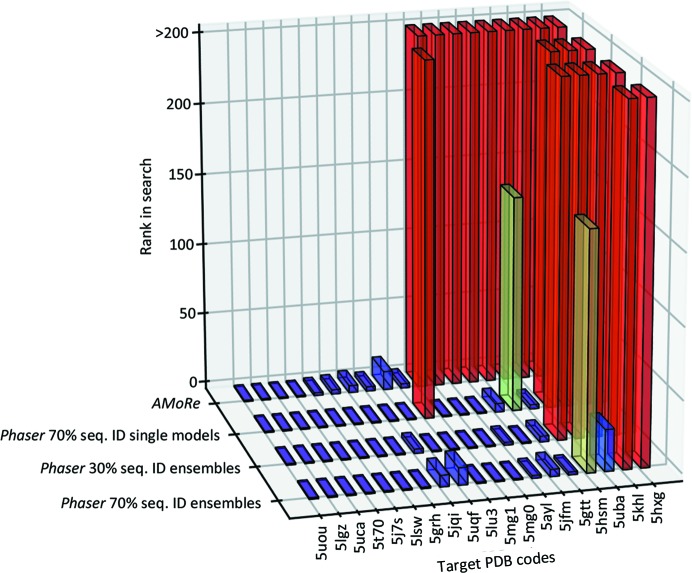
3D bar chart showing the ranking of successful search models using *AMoRe* and *Phaser* with various parameters. The bars are coloured using a rainbow scale where violet indicates a successful search model that has ranked top in the search and red indicates that the highest ranking potentially successful search model lay outside the top 200. As only the top 200 search models are trialled in the MR step, those models which ranked higher than 200 represent unsuccessful searches.

Improving the success rate of *SIMBAD* relies on distinguishing the signal of a correct solution from the noise. It follows that in addition to solving more cases, a successful method will be more likely to rank a good search model highly. Fig. 3 ▸ shows a three-dimensional bar graph of ranking versus method versus PDB code for the 19 test cases known to be solvable. In general, *Phaser* returns more solutions and ranks these solutions higher in the search than *AMoRe*.

## 4. Discussion   

The implementation of the *Phaser* fast rotation search in *SIMBAD* has proved to be significantly better at detecting suitable search models than the version using *AMoRe*. The use of ensembles in *SIMBAD* has helped to further increase the sensitivity of the rotation function when screening the *MoRDa* database. This has allowed us to obtain greater RFZ values for suitable search models and therefore increase the likelihood of a solution and early termination.

**Figure 4:**
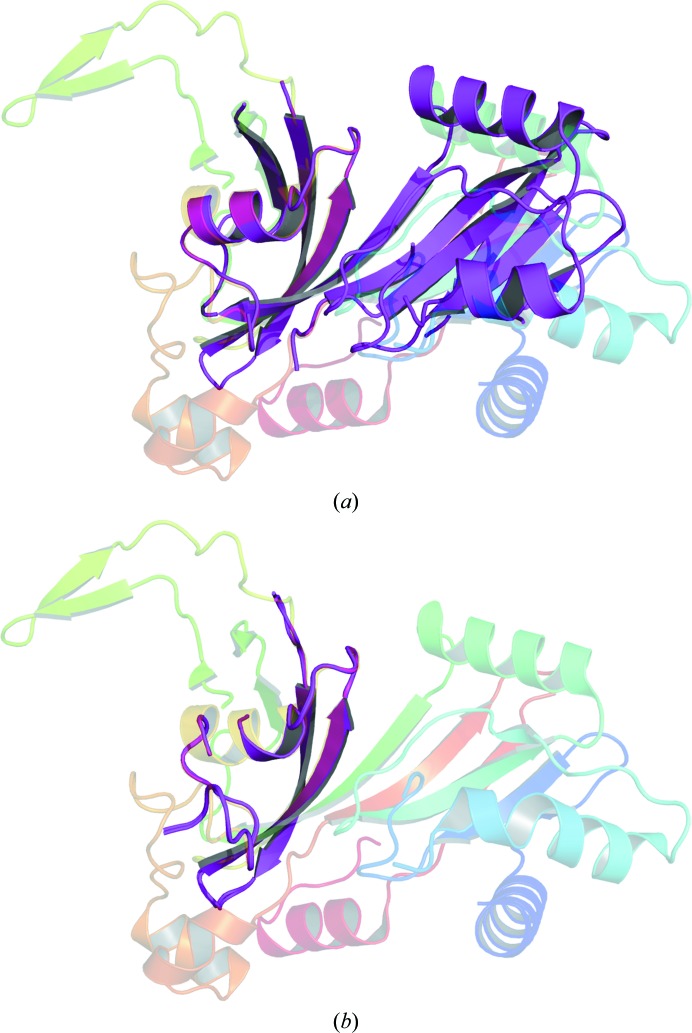
(*a*) The *MoRDa* domain (PDB entry 2i82; magenta) aligned with the crystal structure (PDB entry 5uba; rainbow). (*b*) The *MrBUMP*-derived truncated ensemble (PDB entry 2i82; magenta) aligned with the crystal structure (PDB entry 5uba; rainbow).

PDB entry 5uba was only able to be solved using *Phaser* with 70% predicted sequence identity and ensemble search models. In this instance it seemed that it was not the ensembling but the redefined domain boundaries that allowed the correct search model to be identified. Fig. 4 ▸ shows a comparison between the full *MoRDa* domain and the *MrBUMP*-derived subdomain. In the full *MoRDa* domain we observed that there was some subtle movement between subdomains. This resulted in a greater r.m.s.d. relative to the true structure (1.33 Å) than the subdomain (0.816 Å). Therefore, the smaller (50 residues versus 160 residues), more similar subdomain gave more distinct rotation peaks than the *MoRDa* search models.

The average r.m.s.d. values for the successful search models were all found to be below 1 Å (Fig. 2 ▸). Using a predicted sequence identity of 30% yielded predictions above 1 Å in all cases (Oeffner *et al.*, 2013 ▸), whereas a 70% prediction gave values that were far closer to the true value. Providing better error estimates allowed *Phaser* to give sharper peaks in the log-likelihood scoring, thus making it easier to distinguish good search models. Trialling further estimates may yield a better result, but owing to the length of time that it takes to run *SIMBAD* over the entire *MoRDa* database (>24 h on 100 cores) we decided that the two values for sequence identity were sufficient.

### 4.1. Efficiency improvements   

Whilst moving to this procedure is computationally more expensive, it is tolerably fast when run on a 100-core cluster (2.6 GHz, Intel Xeon E5-2640). With crystallographic software moving onto the cloud (Krissinel *et al.*, 2018 ▸), such clusters are becoming more readily accessible to users. Additionally, the implementation of an early-termination function (LLG > 60 and TFZ > 8 and/or *R* factors of <0.45) allows *SIMBAD* to bypass much of the computational expense if a clear solution is detected. Indeed, applying this to our test set resulted in an ∼35% reduction in average time taken (27.8 to 18.2 h). Additionally, when comparing the nine cases that solved with both *AMoRe* and *Phaser* using single search models, the early-termination function allowed *Phaser* to identify solutions in a lower average time (9.1 versus 10.8 h).

We postulated that increasing the sensitivity of the rotation search may result in even faster run times by way of the early-termination function. *SIMBAD* had previously been modified to include an additional translation step to increase the sensitivity when screening ensembles derived from meta­genomic databases (Simpkin *et al.*, 2019 ▸). A few of the cases that took longer to run were tested with this method and demonstrated significant time reductions. For example, the time taken to find a solution for PDB entry 5uqf decreases to 5 h from the previous 54 h (both performed on a 100-core cluster). Future work will seek to further explore any efficiency advantages that this method might confer.

### 4.2. Future developments   

A key future development is to improve the method by which ensembles are generated for *SIMBAD*. The current strategy uses sequence to identify suitable homologues for ensemble generation, whereas searching the PDB for homologues according to structural similarity (as assessed by programs such as *GESAMT*) may yield better results. So, for example, searching the PDB based on sequence will fail to distinguish between alternate conformations, for example R- and T-states in allosterically regulated enzymes. Processing and ensembling a mixture of such conformational states is likely to hamper structure solution.

The method for creating the ensembles may also be improved upon. In the current version of *MrBUMP*, the ‘seed’ model obtained from the *MoRDa* database is not modified in line with its homologues. This makes sense in the context of sequence-based MR, as the model that is sequentially most similar will be expected to be the most structurally similar. However, in the context of sequence-independent MR this can no longer be assumed. In this instance a better approach would be to modify the ‘seed’ model so that only those loops and side chains common to the homologues remain. This should allow *Phaser* to better estimate the averaged structure factors and subsequently improve the chances of finding the best orientation in the rotation search. Where high-variance ensembles have been generated, truncation might be required in order to find a low-variance core. The use of this type of truncation strategy has been demonstrated to be beneficial by the *AMPLE* project, which exploits the clustering and truncation of thousands of *ab initio*-generated search models for MR (Bibby *et al.*, 2012 ▸; Keegan *et al.*, 2015 ▸).

Another area that we may be able to improve is in the way that search models are sorted prior to the rotation search. The approach presented in this paper (ordering on the basis of MW) is an improvement on the previous method; however, it works best when the target is a monomer. This could be improved by making use of the self-rotation function to identify the presence of noncrystallographic symmetry, obtaining a better estimate of the number of molecules in the asymmetric unit and adjusting the estimated MW of the target accordingly.

We also wish to explore how the search models are selected for the full MR step. By default, the top-ranking 200 search models are taken forward, as this was deemed sufficient to catch the majority of cases where the model is ranked near the top in the search. The number of models tested is a user-configurable parameter and so can be adjusted. However, future research might look at different ways to select these models; for example, searching all solutions that have an RFZ within 10% of the top-ranking search model.

## 5. Conclusions   

The use of the *Phaser* fast rotation search in *SIMBAD*, and the ensemble search models which can therefore be used, each significantly improve the effectiveness of the pipeline. Together with an early-termination function, they allow *SIMBAD* to more readily identify suitable search models in the *MoRDa* database and to identify them more quickly, thereby more efficiently solving a wider range of cases in a sequence-independent fashion.

## Supplementary Material

Supplementary Table S1. DOI: 10.1107/S2059798319015031/rr5186sup1.xlsx

## Figures and Tables

**Table 1:** Results for the 19 test cases where MR solutions were possible The corresponding *MoRDa* search model and Map CC score are shown for the solutions found by each method.